# 

**Published:** 1907-06

**Authors:** 


PUBLISHED MONTHLY. ATLANTA SUBSCRIPTION $1.00

### JOURNAL-RECORD OF MEDICINE

Successor to { Atlanta Medical and Surgical Journal, Established 1855. and Southern Medical Record, Established 1870. } 52d Year

Vol. IX. ATLANTA, GA., JUNE, 1907. No. 3.

